# Development and Characteristics of Protein Edible Film Derived from Pork Gelatin and Beef Broth

**DOI:** 10.3390/polym16071009

**Published:** 2024-04-07

**Authors:** Agnieszka Ciurzyńska, Monika Janowicz, Magdalena Karwacka, Małgorzata Nowacka, Sabina Galus

**Affiliations:** Department of Food Engineering and Process Management, 159c Nowoursynowska St., 02-776 Warsaw, Poland; monika_janowicz@sggw.edu.pl (M.J.); magdalena_karwacka@sggw.edu.pl (M.K.); sabina_galus@sggw.edu.pl (S.G.)

**Keywords:** edible films, beef broth, gelatin, side products

## Abstract

The aim of this work was to develop edible films derived from gelatin and beef broth and to analyze the physical properties of the output products. The presented research is important from the point of view of searching for food packaging solutions that may replace traditionally used plastic packaging. This study’s conceptual framework is in line with the trend of sustainable development and zero waste. This study was conducted to develop a recipe for edible films derived from beef gelatin with gelatin concentrations at 4%, 8%, and 12% enriched with additions of beef broth in amounts of 25, 50, 75, and 100%. Selected physical properties of the output edible films were examined in terms of thickness, swelling in water, opacity, water content, water solubility, structure, and mechanical properties. The conducted research made it plausible to conclude that the addition of broth has a positive effect on the extensibility of the edible films and the other physical properties under consideration, especially on decreasing the film thickness, which was found to vary between 50.2 and 191.6 µm. When gelatin and broth were added at low concentrations, the tensile strength of the films increased, and subsequently decreased; however, an opposite effect was observed for elongation at break. The increased broth concentration caused the film opacity to increase from 0.39 to 4.54 A/mm and from 0.18 to 1.04 A/mm with gelatin concentrations of 4% and 12%, respectively. The water solubility of the gelatin films decreased as a result of the broth addition. However, it was noticed that increasing the content of broth caused the water solubility to increase in the tested films. The mere presence of broth in the gelatin films changed the microstructure of the films and also made them thinner.

## 1. Introduction

An edible coating is a thin layer of edible material, usually made of a biopolymer, in which food is wrapped. An edible film is a structure made of edible material that, before being placed or becoming an integral part of food, is previously processed so that it can properly perform its function. The main difference is that edible coatings are placed on food by, for example, spraying them in liquid form onto the material or immersing the material in a film-forming solution. However, edible films have also been previously formed as durable sheets that are then placed on food [1,2].

Edible films and coatings are used in the food industry mainly to extend the shelf life of food and to make it more attractive to the consumers. They must have appropriate features and functional properties such as barriers for gases occurring in the surrounding environment. If these properties are inappropriate, undesirable substances may accumulate in the structures of raw materials, semi-finished products, or finished products. Disturbing the appropriate proportions in the content of ingredients may launch the processes that will ultimately lead to adverse sensory changes in the product [3,4]. 

Edible films and coatings have been attracting special attention from the scientific community for a long time. This is due to the potential opportunity to replace traditional packaging with packaging made of materials that are suitable for consumption with the product, or those that entail lower costs and harm to the environment upon disposal. From the point of view of solid waste management, biodegradable packaging may prove to be a useful solution if we replace non-biodegradable packaging [5]. 

Edible films and coatings can be made from various types of materials, such as plant or animal hydrocolloids, resins, and lipids, and they can even contain compounds of these raw materials [6]. In order to reduce the cohesion of edible films, which causes them to become hard and brittle, a plasticizer is added to their coating, which removes intermolecular hydrogen bonds [7]. The plasticizers are, for example, saccharides, lipids, and the most commonly used polyols (e.g., glycerol, sorbitol). Films fabricated with the addition of plasticizers are more susceptible to elongation but can withstand less force when stretched. The addition of plasticizers to the edible films also increases their solubility in water. Additionally, some studies have shown that the use of plasticizers such as glycerol causes the water retention to be more effective in gel systems, thereby increasing the flexibility of the entire system [8,9,10]. For the purpose of this manuscript, glycerol was chosen as a plasticizer.

Gelatin is a water-soluble protein mixture derived from the controlled thermal hydrolysis of collagen. Collagen is a protein that is a part of the connective tissue structures of all animals [11]. Defatted bones, bone crumbs, and skins from various animal species constitute the raw materials for the production of gelatin [12]. Gelatin is widely valued in many industries due to its physicochemical properties: thickening, foam formation, gelation, moisture retention, and texture improvement [13]. However, the most valued property of gelatin, especially in the food industry, is that it allows for the creation of reversible gels. There is a method for determining the strength of gels created using gelatin, which was developed in the United States in 1925 by Oscar T. Bloom who, using an invented machine, developed a method for testing gel hardness [14]. Since then, the hardness of gels has been determined in Bloom degrees, which express the force that is needed to cause the deformation of a gel with a specific concentration and temperature. Many researchers have used gelatin to produce biodegradable films due to its good film-forming ability, emulsifying property, and gas barrier property. They have indicated that gelatin can form a strong and flexible film, whereas native gelatin films become fragile if suitable temperature and humidity conditions are not assured. The water resistance of gelatin film is very poor. Therefore, investigations in mixing gelatin with other natural polymers and active ingredients are necessary to improve the functional properties of gelatin [15]. Channa et al. [16] obtained environmentally friendly edible films and showed that adding food color to gelatin–cornstarch-based films enhanced the resistance to moisture, mechanical characteristics, and thermal stability. Tran et al. [17] indicated that the disadvantages of gelatin films may be alleviated by mixing gelatin with biofilm-forming agents, which provide a good barrier of lipids, oxygen, and carbon dioxide.

This work aimed to obtain edible films using gelatin as a biodegradable plastic and cheap material for the production of bio-based packaging materials. One of the possibilities for modifying the properties of the edible film obtained from gelatin is to add a fat emulsion to it, e.g., beef broth. The addition of beef broth is expected to affect selected physical properties of the obtained coatings, making the product more attractive and different from the standard biodegradable packaging. So far, no studies have analyzed how adding beef broth to film-forming solutions may change their properties, but there are many studies that have aimed at examining how the properties of edible films change after various types of fat emulsions have been added to them. It has been proven that the addition of fat significantly improves the barrier properties of the films, especially in terms of moisture protection, which is due to the hydrophobic nature of lipids. Yang and Paulson [18] proved that the addition of fat to gellan films causes mechanical and optical properties to deteriorate. An inverse relationship was observed in the research conducted by Galus and Lenart [19], where adding fat emulsion to whey coatings slightly improved the mechanical properties of those coatings. The research at issue suggests that the effect of fat emulsion on the properties of the edible films and coatings is ambiguous and largely depends on the material used for testing purposes.

## 2. Materials and Methods

### 2.1. Research Material

The research material consisted of the protein films derived from the following ingredients (Table 1).

### 2.2. Preparation of Edible Protein Films

The solutions used for producing the edible films (control samples) were obtained through dissolving pork gelatin (Gelita AG, Eberbach, Germany) in distilled water. Appropriate amounts of pure gelatin were measured and poured into three different beakers. The beakers were then filled in with distilled water to reach a volume of 200 mL. Three solutions were heated up to 60 °C by means of the RCT basic magnetic stirrers (IKAPoland Sp. z o.o., Warsaw, Poland) rotating at 300 rpm, and the solutions were kept at such a temperature for 30 min, being constantly stirred. The next step was to add glycerol (Avantor Performance Materials Poland S.A., Gliwice, Poland) to each beaker in the amount equivalent to the gelatin concentration at 50%, cool the solutions down to 50 °C, and mix again for 30 min by means of the rotating magnetic mixer at 300 rpm, maintaining the set temperature. The solutions prepared in this way were poured onto plastic dishes in the amount equivalent to 10 mL of the film-forming solution to obtain a constant film thickness. Then, the solutions were dried for 36 h at 30 °C in the universal dryer with the electronic temperature controller type SUP-65 WG (WAMED, Warsaw, Poland). After this time, the gelatin films were peeled off from the Petri dishes, conditioned at the temperature of 25 °C and relative humidity of 50% for 48 h, and then were ready for analysis.

**Figure 1:**
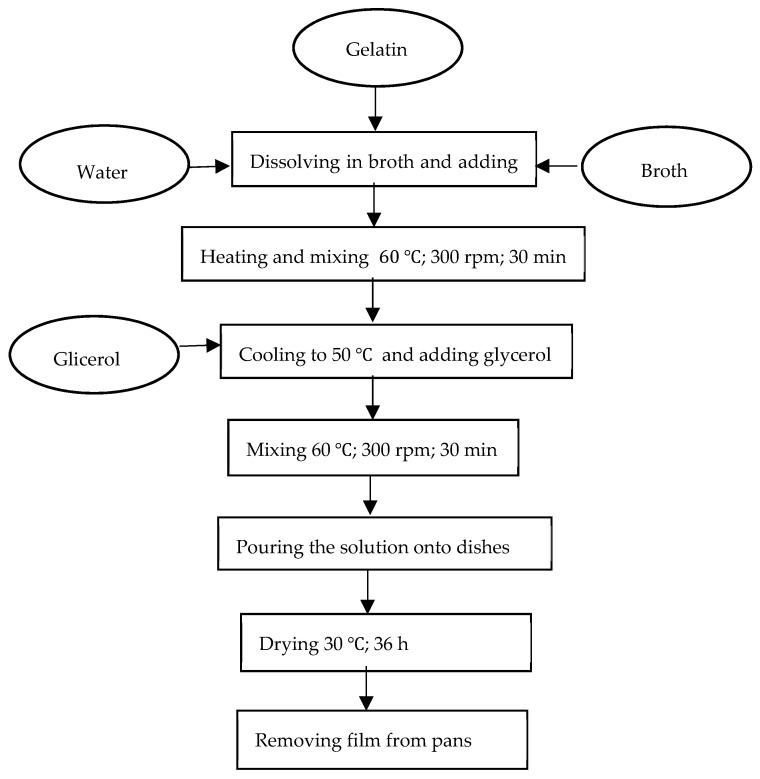
Technological diagram for preparing the edible films for the research test purpose.

The preparation of the edible films for test sampling purposes took place (Figure 1) after all the analytical determinations had been made to the extent of the control sample (without broth). The activities began with the preparation of the film-forming solution. Appropriate amounts of food gelatin (Gelita AG, Eberbach, Germany), previously measured on scales, were poured into beakers (Table 1). Then, the previously prepared broth (Winiary, Nestlé Polska S.A., Kalisz, Poland) was poured into the beakers in the amount shown in Table 1 and, if necessary, topped up with distilled water to reach the target volume of 200 mL. Then, all the solutions were heated up to 60 °C for 30 min, stirring constantly by means of the RCT basic magnetic stirrers [IKA Poland Sp. z o.o., Warsaw, Poland] (300 rpm). Next, the solutions were cooled down to 50 °C, and then glycerol (Avantor Performance Materials Poland S.A., Gliwice, Poland) was added in the amount corresponding to 50% of the gelatin content in a given solution and the solution temperature was maintained on the magnetic mixer rotating at a constant speed of 300 rpm for 30 min. The solutions prepared in this way were poured onto plastic dishes and dried at 30 °C for 36 h in the universal dryer by means of the electronic temperature controller type SUP-65 WG (WAMED, Warsaw, Poland). Upon completion of that procedure, the films were removed from the dishes, conditioned at the temperature of 25 °C and relative humidity of 50% for 48 h, and then used for analysis.

### 2.3. Analytical Methods

#### 2.3.1. Film Thickness

The thickness of the films was determined by means of the ProGage thickness gauge (Thwing-Albert, West Berlin, NJ, USA) with an accuracy of 1 μm. The measurement was performed in 10 repetitions for each type of film.

(1) $$\bar{\sigma }=\sum_{i=0}^{n}\sigma_{i},$$

where: $\bar{\sigma }$—average sample thickness; $\sigma_{i}$—thickness value for measurement *i*, (μm); *n*—number of measurement repetitions.

#### 2.3.2. Swelling in Water

In order to determine the swelling, samples of 20 × 20 mm dimensions were cut from the coatings and weighed on the Adventurer AX223 analytical scale (OHAUS CORPORATION, Parsippany, NJ, USA) with an accuracy of 0.0001 g. Then the films were immersed in approximately 25 mL of distilled water at a temperature of 25 °C. The samples were kept in water for 2 min, then removed and dried on filter paper. The next step was to measure the weight of the coatings again. The measurement was performed in triplicate for each type of coating [20].

(2) $$P=\frac{m_{m}-m_{s}}{m_{s}},$$

where: *P*—swelling, (%); *m_m_*—wet sample weight, (g); *m_s_*—dry sample weight, (g).

#### 2.3.3. Opacity

Opacity measurement was performed by means of the Thermo Scientific UV–visible spectrophotometer Evolution 200 series (Fisher Scientific International Inc., Waltham, MA, USA). The samples were placed on the feeder by means of the magnet, and absorbance was measured at the wavelength of 600 nm in ten repetitions for each type of sample [21].

(3) $$O=\frac{A_{600}}{\bar{\sigma }},$$

where: *O*—opacity, (1∙mm^−1^); *A*_600_—absorbance at wavelength 600 nm; $\bar{\sigma }—$average sample thickness, (mm).

#### 2.3.4. Water Content

The water content was determined based on the determination of the dry matter content, which was determined using the gravimetric method. The samples, measuring 20 × 20 mm, were cut from the edible films, placed in the measuring cups, and then weighed by means of the Adventurer AX223 analytical scale (OHAUS CORPORATION, Parsippany, NJ, USA) with an accuracy of 0.0001 g. The prepared samples were dried in the universal dryer with the electronic temperature controller SUP-65 WG (WAMED, Warsaw, Poland) for 24 h at 105 °C. Next, the sample bottles were cooled down in the CaCl_2_ desiccator and weighed again. The determination of the relevant parameters was performed in triplicate for each type of sample [20].

(4) $$u=\frac{m_{1}-{\;m}_{2}}{m_{1}}*100\%,$$

where: *u*—water content, (%); *m*_1_—sample weight before drying, (g); *m*_2_—sample weight after drying, (g).

#### 2.3.5. Solubility in Water

Samples of 20 × 20 mm dimensions were cut from the edible films, placed in the measuring vessels, and then weighed by means of the Adventurer AX223 analytical scale (OHAUS CORPORATION, Parsippany, NJ, USA) with an accuracy of 0.0001 g. Then the vessels with the samples were placed in the universal dryer with the electronic temperature controller type SUP-65 WG (WAMED, Warsaw, Poland) for 24 h at a temperature of 105 °C, and then were cooled down in the CaCl_2_ desiccator and weighed again. Next, the samples were inundated with 25 mL of water and left for 24 h, being stirred occasionally. The samples were subsequently removed from the water and dried by means of the filter paper. Then they were weighed again on the analytical scale and dried again at 105 °C for 24 h. After drying, the samples were cooled down in the CaCl_2_ desiccator and finally weighed again by means of the analytical scale. The measurement was performed in triplicate for each type of sample [22].

(5) $$R=\frac{m_{o}-m_{r}}{m_{o}}*100\%,$$

where: *R*—solubility in water, (%); *m_o_*—dry weight of the sample before dissolution, (g); *m_r_*—dry weight of the sample after being kept in water, (g).

#### 2.3.6. Structure

The structure was analyzed based on the photographs taken by means of the TM-3000 HITACHI scanning electron microscope (Hitachi High-Technologies Corporation, Tokyo, Japan). The samples were prepared by cutting squares of 5 × 5 mm from the film, and fixing them on a metallic cylindrical support, which was attached to a measuring table using double-sided tape PELCO with a diameter of 9 mm (Pik Instruments Sp. z o.o., Piaseczno, Poland); they were then covered with gold using gold coater model Sputtercoater 108 auto (Cessington Scientific Instruments, Watford, UK). No particular film preparation was necessary. The samples prepared in this way were placed in the measuring chamber of the microscope and photos of the cross-section and the surface of the coatings were taken. The photos of the cross-sections were taken at 800, 1000×, and 1200× magnification.

#### 2.3.7. Mechanical Properties

Mechanical properties were measured by means of the TA-XT2i texture meter (Stable Microsystems, Great Britain, UK) and the Texture Expert computer program. The measurement procedure was performed in ten repetitions for each type of coating. The samples, measuring 25 × 100 mm, were developed from the films, and then the thickness of each sample was measured using the ProG-age thickness gauge (Thwing-Albert, West Berlin, NJ, USA) at three random places on the sample. The arithmetic mean of the three measurements was calculated and recorded as the sample thickness. The samples were then placed between the jaws of the texturometer, which were suspended at a constant distance of 25 mm away from each other. The jaws moved apart at a constant speed of 1 mm/s, stretching the sample until it burst. The computer program recorded the measurement of the maximum force needed to tear the coating as well as the distance to which the coating was stretched at the moment of tearing. Based on the obtained results, two discriminants for the tensile strength of the film were calculated:

(6) $$TS=\left(\frac{F_{max}}{A}\right),$$

where: *TS*—tensile strength, [MPa]; *F_max_*—tearing force needed to rip the coating, [N]; *A*—cross-sectional area of the sample before the tensile test, [mm^2^], where:

(7) $$E=\left(\frac{∆l}{l_{o}}\right)$$

where: *E*—relative elongation, [%]; ∆*l*—elongation of the sample at which the coating ruptured, [mm]; *l_o_*—initial length of the sample before the tensile test, [mm].

#### 2.3.8. Statistical Analysis

The obtained results were subjected to the statistical analysis in the form of a two-factor analysis of variance conducted in the Statistica program. In addition, the selected determinations were additionally subjected to the Pearson’s correlation analysis also conducted in the Statistica program.

## 3. Results and Discussion

### 3.1. The Influence of Gelatin and Beef Broth Concentration on the Thickness of Edible Protein Films

The thickness-related results for the gelatin films and gelatin–broth films are presented in Table 2. When comparing the edible films differing only in the content of broth and containing the same concentration of gelatin in their composition, it was noticed that the films without broth (lower mass of dry substance/cm^2^) are characterized by a greater thickness than the films that contained broth. Similar results were obtained by Galus and Lenart [20] who reported that the addition of fat emulsion to the edible films reduced the thickness of those films. When analyzing the effect of varied amounts of broth on the thickness of the film, it is difficult to notice a clear relationship, which indicates a linear dependence of the change in thickness on the change in the broth content in the tested films. The effect of the gelatin concentration on the target film thickness is more explicit because the amount of gelatin in the film is directly proportional to the film thickness. Cui et al. [23] also showed that the composite film’s thickness grew concurrently with the increase in the number of nanoparticles.

**Figure 2:**
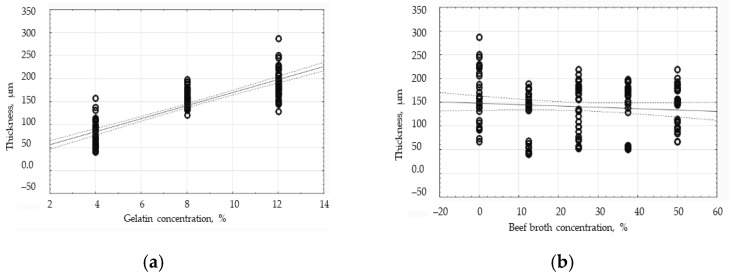
The influence of the gelatin concentration (**a**) and beef broth concentration (**b**) on the thickness of the edible protein films.

The two-way analysis of variance has shown that, at the 95% confidence level, there was a statistically significant effect (*p* < 0.001) of gelatin concentration, broth concentration, and the combination of those two factors on the thickness of the edible films. In order to determine the correlation between the tested factors and the thickness of the protein coatings, correlation analysis was carried out. The analysis of the obtained results has shown a strong positive correlation between the gelatin concentration and the coating thickness at the level of the Pearson’s correlation coefficient of 0.8577 (*p* < 0.05). However, the correlation between the broth concentration and the thickness of the tested coatings has turned out to be weak and negative since the value of the correlation coefficient equaled −0.0928. The results of the correlation analysis are presented in Figure 2. The graph of the influence of the gelatin concentration on the thickness of the edible films (Figure 2a) shows a directly proportional relationship between those two variables. Figure 2a exhibits more consistent results than Figure 2b, indicating a much greater effect of the gelatin concentration than the broth concentration on the thickness of the edible films. A large discrepancy has been observed in the results within one group in the graph presented in Figure 2b. In this graph, within one broth concentration, the results in respect of the films containing varied concentrations of gelatin are included to support the conclusion that the concentration of gelatin is decisive for the thickness proven by the edible films.

### 3.2. The Influence of Gelatin and Beef Broth Concentration on the Opacity of Edible Protein Films

**Figure 3:**
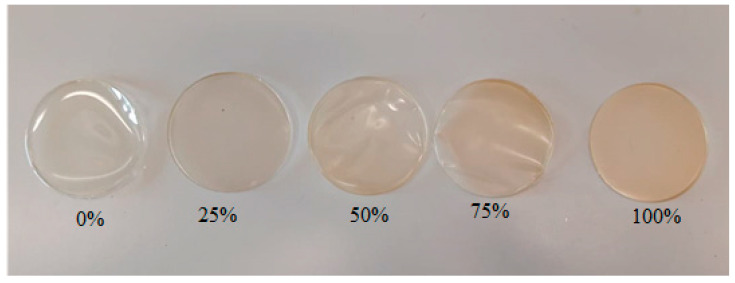
The edible films with 12% gelatin concentration and broth concentration at 0%, 25%, 50%, 75%, and 100%.

Figure 3 shows the photos of the edible films that contain 12% gelatin and various broth concentrations. The visual assessment has allowed us to determine the differences between the gelatin film and the gelatin–broth film. The gelatin films had a uniform structure on both sides, similar gloss, relative stiffness, and excessive bending could result in breakage. However, the gelatin–broth films had a different texture on each side. On the aired side, the film was more matte and smoother than on the side adjacent to the pan.

The visual assessment of the film has allowed us to arrive at the conclusion that the films become less and less transparent with the increase in the broth concentration. The gelatin films have turned out to be very transparent. That relationship is particularly visible when comparing the sample containing only gelatin (0% broth) and the sample containing 100% broth. The visual assessment outcome coincides with the results of the film opacity analysis (Table 3). Cui et al. [23] also indicated that the thickness of the coating was a very important element as far as inhibiting light transmission was concerned. The opacity of the film increases with the increase in the amount of nanoparticles, which may explain the higher opacity of the gelatin–broth films as compared to the gelatin films. The two-way analysis of variance has shown that the gelatin concentration, broth concentration, and the combination of both factors have a significant impact on the opacity value proven by the films (*p* < 0.001; α = 0.05).

**Figure 4:**
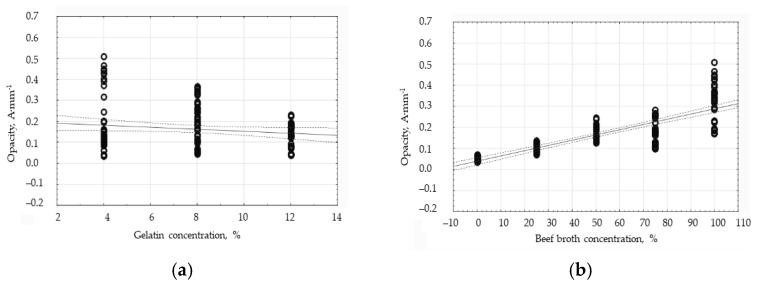
The influence of the gelatin concentration (**a**) and broth concentration (**b**) on the opacity of the edible protein films.

The Pearson’s correlation analysis has shown that the gelatin concentration and the broth concentration differently affect the opacity value of the edible films. The results are shown in Figure 4. In the case of the effect of the broth concentration on the opacity value (Figure 4b), the correlation factor has proven to be as high as 0.8272. That evidences a strong and positive correlation. Moreover, it is worth noting that the discrepancy in the results within the respective broth concentration groups is greater, the higher the broth concentration in the edible films. The standard deviation therefore varies to a greater extent in the higher broth concentration groups concurrently with the changes in the gelatin concentration. Based on those results, it is plausible to conclude that for the films containing the higher broth concentration, the gelatin concentration has a greater impact on the opacity value, and the effect of the gelatin concentration decreases when the broth concentration decreases in the films. The correlation analysis for the gelatin concentration and opacity has resulted in the value of 0.1500, which indicates a weak and negative correlation. Having analyzed the graph for this relationship (Figure 4a), the discrepancy in the results within one group of the gelatin concentrations has been found to be smaller the higher the gelatin concentration in the tested films. The standard deviation for all the groups of results equals 59.52% on average. However, it is also the case that there is a clear difference in the range of results within the respective groups. In the 4% gelatin concentration group, the standard deviation is as high as 80.72%, while in the 8% and 12% groups, it is 52.99% and 44.84%, respectively. Therefore, the standard deviation for the respective groups is lower, the higher the gelatin concentration. That indicates that for the films containing the lower gelatin concentration, the broth concentration has a greater impact on the opacity value, and the effect of the broth concentration decreases with the increase in the gelatin concentration. The gelatin concentration has an opposite effect on the opacity value of the edible films as compared to the broth concentration.

### 3.3. The Influence of Gelatin and Beef Broth Concentration on the Swelling of Protein Edible Films in Water

Swelling of the edible films in water has been tested (Table 4). The increase in the swelling value in water has been found to be concurrent with the increase in the gelatin concentration in the gelatin films without the addition of beef broth. However, in the case of the gelatin–broth films, the opposite effect has been observed since the broth concentration slightly affects the water swelling of the edible films. The comparison of the results for the gelatin and gelatin–broth films within one gelatin concentration indicates that both types of films differ significantly. The high standard deviation for each of the tested types of foil may be the result of uneven drying of the coatings on the surface of the pan onto which the film-forming solution was poured, which could have resulted in obtaining a different structure of the samples that swelled to varying degrees.

In order to interpret the results of all types of films, only gelatin–broth films underwent the statistical analysis. The two-way analysis of variance, taking into account the gelatin and gelatin–broth films, has proven that the delivered swelling values in water are not influenced by the concentration of broth in the films. However, the gelatin concentration and the combination of the effects of the gelatin concentration and broth concentration have a significant impact on the swelling of the film in water (*p* < 0.01; α = 0.05) (Table 5).

However, if only the gelatin–broth films had been subjected to the two-way analysis of variance, the results would have shown that the swelling of the films in water was influenced only by the concentration of gelatin in the films, while the concentration of the broth and the combination of both factors was insignificant (*p* < 0.01; α = 0.05) (Table 6). The results of the statistical analysis indicate that the mere presence of broth in the edible films affects swelling in water, while changing the concentration of broth in the films does not significantly affect swelling in water.

### 3.4. The Influence of Gelatin and Beef Broth Concentration on the Water Content of Protein Edible Films

The water content in the edible films is shown (Table 7). The films derived from the 4% gelatin and 0% broth had the highest water content. Taking into account the results for the gelatin–broth films, for each broth concentration, the highest water content was found in the films containing 8% gelatin, while in the case of the gelatin films, the water content decreased with the increase in the gelatin content in the tested films. Similarly, Susilawati et al. [24] obtained the water content decrease in the composite films with the concurrent increase in the concentration of chitosan. The two-way analysis of variance has shown that the gelatin concentration, broth concentration, and the combination of both factors have a statistically significant effect on the water content in the edible films (*p* < 0.001; α = 0.05).

**Figure 5:**
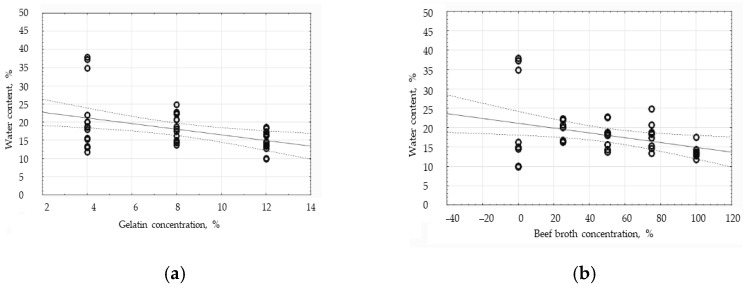
The influence of the gelatin concentration (**a**) and broth concentration (**b**) on the water content of the edible protein films.

The Pearson’s correlation analysis has shown a statistically significant but weak and negative relationship between the gelatin concentration and water content in the coatings, since the correlation coefficient equaled −0.4136. Having analyzed the graph illustrating that relationship (Figure 5a), all three groups of measurements have proven the results to be relatively concentrated, except for the results in the “4% gelatin” group; the deviation data belong to the films without broth. That group of results gives the trend line a downward-sloping shape. In the case of the graph of the relationship between the water content and the broth concentration (Figure 5b), a slightly weaker but also negative relationship has been observed. The correlation coefficient is −0.3546. The results belonging to the gelatin films are characterized by a large scatter, so within those results, the gelatin concentration significantly affects the water content. However, for the gelatin–broth films, the data are much more concentrated, so the gelatin concentration has less influence on the result. The water content decreases with the increase in the broth concentration in the edible films. The presented data analysis has allowed for the conclusion that the addition of broth to the edible films changes how the film components bind water in the gelation process.

### 3.5. The Influence of Gelatin and Beef Broth Concentration on the Water Solubility of Protein Edible Films

The solubility of the edible films in water has been tested. In the case of the film with the concentration of 4% gelatin and 0% broth, the dissolution of the edible film was so advanced that no further mass measurement was possible, so the dissolution was assumed to be complete, or “100%” (Table 8). In the statistical analysis regarding that determination, it was decided to omit that particular result. Lu et al. [15] indicated that the water resistance of the gelatin film was poor, and samples in contact with water may have dissolved or disintegrated. The analysis of Table 8 has allowed us to conclude that the gelatin concentration does not significantly affect the solubility of the edible films. The addition of broth decreases the solubility of the samples as compared with the gelatin films without broth. In most cases, the water solubility increases as the broth content increases in the edible films. The two-way analysis of variance has shown that the gelatin concentration, broth concentration, and the combination of those two factors have a statistically significant effect on the water solubility of the tested edible films (*p* < 0.05; α = 0.05).

**Figure 6:**
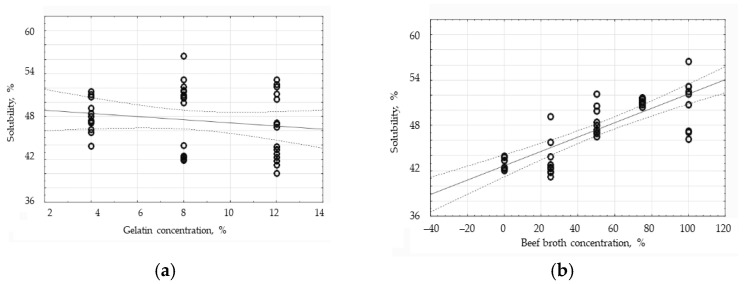
The influence of the gelatin concentration (**a**) and broth concentration (**b**) on the water solubility of the protein edible films.

The conducted Pearson correlation analysis has proven that there is a relatively strong, positive relationship between the broth concentration and the water solubility, since the correlation coefficient equaled 0.7999 (Figure 6b). The solubility of the edible films is directly proportional to the concentration of the broth. The dependence of the water solubility on the gelatin concentration of the edible films has turned out to be negative, weak, and practically statistically insignificant because the correlation coefficient equaled only −0.1275. Having analyzed the chart (Figure 6a), the group of the results with ‘8% gelatin’ has indicated to be characterized by the greatest dispersion of data; in this group, the films with varied broth concentrations are the most diverse in terms of solubility in water. A slightly lower spread of results has been observed in the ‘12% gelatin’ group. The smallest data dispersion occurs in the ‘4% gelatin’ group; the standard deviation in this group is 4.56% on average.

### 3.6. The Influence of Gelatin and Beef Broth Concentration on the Mechanical Properties of Edible Protein Films

**Figure 7:**
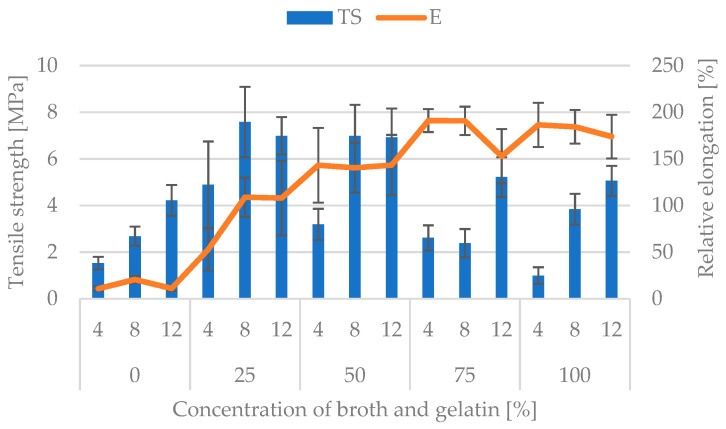
Average values of the tensile strength (TS) and relative elongation (E) for the respective gelatin and broth concentrations.

Benbettaïeb et al. [25] indicated that the mechanical properties of the edible films were very important due to their influence on product properties. Gelatin, as an animal protein, is not structurally stable, but introducing a plasticizer (glycerol) into the protein matrix may improve such properties [26]. Gumus et al. [27] indicated that the soft and tough edible film based on fish gelatin with the addition of red wine lees and carrageenan, with low TS and high EAB values, proved to have great potential as food packaging materials. The tensile strength (TS) and relative elongation (E) of the edible films have been tested (Figure 7). The tensile strength has been found to gradually increase with the concurrent addition of broth at the level of 25%, and to decrease when the broth content increases in the tested films. A similar relationship has been observed by Fang et al. [28] as a result of having analyzed the effect of soybean oil on the tensile strength of the films containing whey protein. The study has shown an initial increase in strength that subsequently decreases when the soybean oil content increases in the films. Vargas et al. [29], having examined the effect of oleic acid on the properties of the chitosan coatings, also proved a gradual increase in the tensile strength of the coatings with the concurrent addition of fat at the level of 10%, followed by a decrease in strength when the fat content in the coatings was increased. Wang and Zheng [30] have shown that the addition of 1% glutaminase increases the strength of the gelatin film but decreases it when its concentration of glutaminase increases to a greater extent. Benbettaieb et al. [25] and Mohammadi et al. [31] proved that increasing the percentage share of gelatin in the gelatin–chitosan films increased the tensile strength of the edible film. The value of the tensile strength, within the respective groups of broth concentrations, increases the higher the gelatin concentration becomes. That trend is characteristic for all the studied cases, but there is a noticeable deviation from it for the films containing the broth concentrations at 50% and 75%. In those cases, the 8% gelatin concentrations reach values slightly higher than the 12% gelatin concentrations, but those values are very similar. However, the value of the relative elongation (E) is the highest for the films containing 100% broth and gradually decreases when the concentration of broth in the edible films decreases. Only the films with 4% gelatin concentration and 25% broth concentration deviate significantly from that rule, which may be due to the irregular thickness of those films. Within the respective broth concentrations, the relative elongation varies slightly for the varied gelatin concentrations. The two-way analysis of variance has shown that both the broth concentration, gelatin concentration, and the combination of both factors influence, to a statistically significant extent, both the tensile strength and the value of the relative elongation of the tested edible films (*p* < 0.05; α = 0.05).

**Figure 8:**
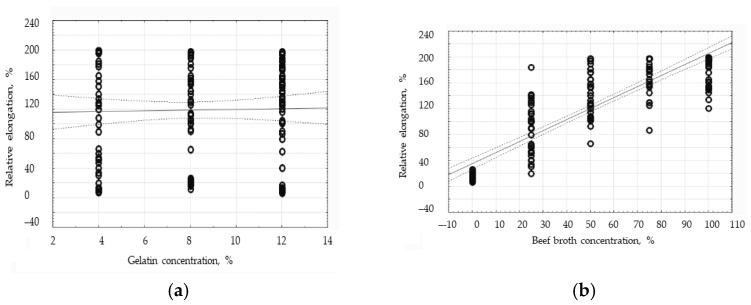
The influence of the gelatin concentration (**a**) and broth concentration (**b**) on the average values of the elongation (E) of the protein edible films.

In order to examine the effect of the gelatin and broth concentrations on the TS and E factors, the correlation analysis has been carried out to show that the broth concentration is strongly positively correlated with the value of the relative elongation demonstrated by the edible films, since the correlation coefficient equaled 0.8782. Having analyzed the graph illustrating that correlation (Figure 8b), the dispersion of the results in the broth concentration groups has been indicated to belong to the gelatin–broth films and to be significant; therefore, within those groups, the gelatin concentration largely affects the value of the relative elongation. However, the scatter of the results in the group of the gelatin films is small, so within that group, the gelatin concentration has a small impact on the relative elongation of the film (Figure 8b). The correlation analysis of the relative elongation and the gelatin concentration has shown that there is no statistically significant relationship between those factors, since the correlation coefficient equaled 0.0219. It has been observed that all the gelatin concentrations have reached similar values for the relative elongation of the tested edible films (Figure 8a).

**Figure 9:**
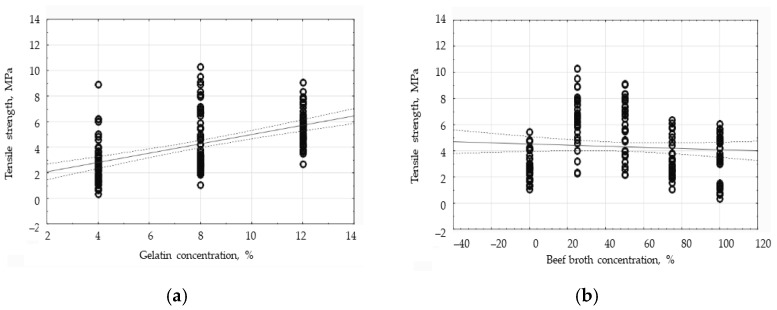
The influence of the gelatin concentration (**a**) and broth concentration (**b**) on the average values of the tensile strength (TS) of the protein edible films.

The correlation analysis of the tensile strength and broth concentration has shown that there is no statistically significant relationship between those two factors, since the correlation coefficient equaled −0.0727. In the graph showing that relationship (Figure 9b), the group of the results belonging to the control sample has been observed to deviate significantly from the trend line created by the concentration results characteristic of the gelatin films, i.e., those not containing broth (Figure 9a). If only the gelatin–broth films had been analyzed, the result would have indicated a stronger negative correlation. The correlation analysis of the tensile strength and the gelatin concentration has proven that there is a statistically significant, positive relationship between those two factors, since the correlation coefficient equaled 0.5341. In the figure illustrating that relationship (Figure 9a), the dispersion of results within the respective concentration groups has been found to be significant but the largest dispersion can be seen in the ‘8% gelatin’ group, which results in the correlation coefficient being underestimated.

### 3.7. The Influence of Gelatin and Beef Broth Concentration on the Structure of Edible Protein Films

**Figure 10:**
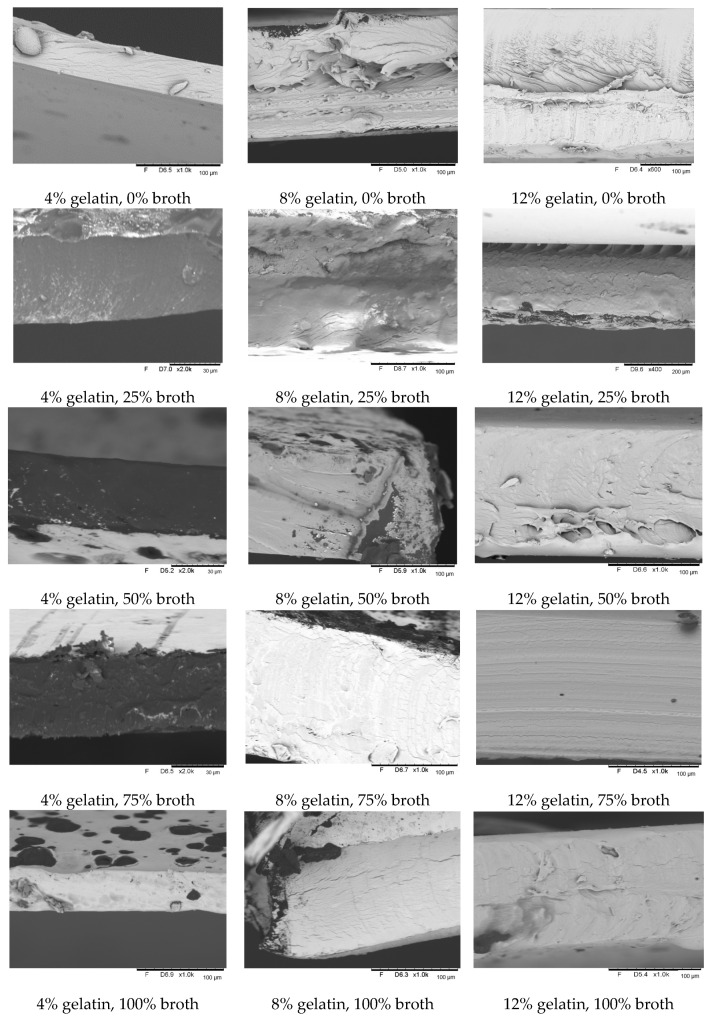
Cross-sectional structures of the tested gelatin and gelatin–broth edible films.

The structure of the gelatin–broth edible films has been examined by means of scanning electron microscope (Figure 10). Having analyzed the cross-sectional photos of all the films and used the scale exhibited in each photo, the thickness of the tested films has been found to be greater, the higher the concentration of the gelatin in the film. That observation coincides with the previously performed measurement of the film thickness. Given the photos, the positive correlation between the gelatin concentration and the thickness of the tested edible coatings has been observed during the thickness measurement procedure. When comparing the photos taken with the gelatin films and gelatin–broth films, some differences in the appearance have been spotted. The films that contain broth seem to create a much more ordered structure than the films that do not contain broth. In the group of films containing no broth, the respective layers of the tested films were arranged unevenly, while in the case of the films containing broth, the respective layers were arranged evenly, one on top of the other. The films that contain broth also have a much more compact structure, and imperfections in the structure are less likely to be noticed than in the case of the films that do not contain broth. All of that may have an impact on the differences in properties that have been studied in this work.

## 4. Conclusions

The work has attempted to develop the composition of the edible gelatin films with the addition of beef broth and examined their selected physical properties. The food industry uses very large amounts of plastic for food packaging purposes. The development of biodegradable coatings derived from pork gelatin with the addition of beef broth, which affects the physical properties of the packaging and makes the packaging more attractive, is in line with the trend of sustainable development and the zero waste conceptual framework.

The content of the broth has significantly reduced the thickness of the edible films and changed the microstructure of the films to a more compact one, too. The higher broth content has resulted in the increase in the opacity and water solubility of the edible films under consideration. Increasing the broth concentration up to 25% in the edible films increases the tensile strength, and then the strength decreases when the concentration decreases. The films that contain a higher concentration of broth are more stretchable. Increasing the concentration of gelatin in the edible films has increased their thickness and tensile strength.

In order to fully determine the applicability of those coatings, storage tests should be carried out in various conditions of the surrounding environment. The organoleptic assessment will also provide for valuable information on the possibility of using the coatings as edible packaging.

## Figures and Tables

**Table 1:** Composition of the edible gelatin films with and without the addition of beef broth.

Solution Number	Amount of Broth [% vol. Solution]	Gelatin Concentration [%]	Glycerol Concentration [%]
1	0	4	2
2		8	4
3		12	6
4	25	4	2
5		8	4
6		12	6
7	50	4	2
8		8	4
9		12	6
10	75	4	2
11		8	4
12		12	6
13	100	4	2
14		8	4
15		12	6

**Table 2:** Average values of the thickness of the gelatin–broth edible films [μm].

Amount of Broth (% vol. Solution)	Gelatin Concentration [%]	Film Thickness [μm]	Weight d.s./cm^2^ [g d.s./cm^2^]
0	4	^c^ 107.23 ± 27.0	0.0100
	8	^ef^ 162.41 ± 13.8	0.0210
	12	^i^ 235.17 ± 22	0.0250
25	4	^a^ 50.2 ± 9.5	0.0113
	8	^d^ 141.9 ± 5.5	0.0212
	12	^f^ 167.2 ± 14.2	0.0258
50	4	^b^ 78.2 ± 18.8	0.0121
	8	^de^ 151.2 ± 18.4	0.0226
	12	^h^ 191.6 ± 13.9	0.0265
75	4	^a^ 54.0 ± 3.1	0.0129
	8	^fg^ 174 ± 17.9	0.0234
	12	^f^ 168 ± 20.3	0.0271
100	4	^bc^ 92.5 ± 15.7	0.0137
	8	^de^ 152.1 ± 4.5	0.0242
	12	^gh^ 188.6 ± 12.7	0.0277

The letters a–i in the upper index indicate belonging to homogeneous groups, between which no statistically significant differences were found (*p* < 0.05).

**Table 3:** Average opacity values for the gelatin (0% broth) and gelatin–broth edible films [A/mm].

Opacity ± Standard Deviation [A/mm]			
Broth Concentration [%]	Gelatin Concentration [4%]	Gelatin Concentration [8%]	Gelatin Concentration [12%]
0	^a^ 0.40 ± 0.01	^a^ 0.353 ± 0.01	^a^ 0.18 ± 0.00
25	^b^ 1.87 ± 0.01	^bc^ 0.79 ± 0.01	^b^ 0.56 ± 0.02
50	^e^ 2.13 ± 0.04	^g^ 1.31 ± 0.03	^d^ 0.71 ± 0.01
75	^c^ 2.14 ± 0.01	^h^ 1.45 ± 0.02	^ef^ 1.05 ± 0.01
100	^j^ 4.54 ± 0.05	^i^ 2.21 ± 0.03	^fg^ 1.04 ± 0.02

The letters a–j in the upper index indicate belonging to homogeneous groups, between which no statistically significant differences were found (*p* < 0.05).

**Table 4:** Average swelling values in water [%] of edible gelatin films (0% broth) and gelatin–broth films.

Swelling in Water ± Standard Deviation [%]			
Broth Concentration [%]	Gelatin Concentration [4%]	Gelatin Concentration [8%]	Gelatin Concentration [12%]
0	^a^ 124.9 ± 80.34	^abc^ 223.01 ± 113.26	^d^ 482.29 ± 287.51
25	^cd^ 372.47 ± 30.24	^abc^ 276.93 ± 20.09	^abc^ 239.53 ± 113.24
50	^bcd^ 326.69 ± 57.94	^abc^ 244.02 ± 66.32	^ab^ 162.00 ± 15.34
75	^cd^ 362.02 ± 94.02	^ab^ 188.58 ± 35.31	^ab^ 195.88 ± 5.47
100	^bc^ 309.55 ± 94.60	^abc^ 209.66 ± 56,62	^ab^ 174 ± 13.13

The letters a–d in the upper index indicate belonging to homogeneous groups, between which no statistically significant differences were found (*p* < 0.05).

**Table 5:** The statistical analysis of the influence of the broth and gelatin concentration on swelling in water—the two-way analysis of variance (research and control samples).

	Degrees of Freedom	SS	MS	F	*p*-Value
Broth concentration [%]	4	27.699	6925	1.3578	0.272763
Gelatin concentration [%]	2	55.749	27.875	5.4657	0.009676
Broth concentration [%] and gelatin concentration [%]	8	139.997	17.500	3.4313	0.006755

**Table 6:** The statistical analysis of the influence of the broth and gelatin concentration on swelling in water—the two-way analysis of variance (only research samples).

	Degrees of Freedom	SS	MS	F	*p*-Value
Broth concentration [%]	3	21.896	7299	1.9648	0.146201
Gelatin concentration [%]	2	146.230	73.115	19.6826	0.000009
Broth concentration [%] and gelatin concentration [%]	6	10.016	1669	0.4494	0.838217

**Table 7:** Average values of water content [%] in the gelatin (0% broth) and gelatin–broth edible films.

Water Content ± Standard Deviation [%]			
Broth Concentration [%]	Gelatin Concentration [4%]	Gelatin Concentration [8%]	Gelatin Concentration [12%]
0	^f^ 36.69 ± 1.53	^bcd^ 15.25 ± 0.83	^a^ 10.06 ± 0.10
25	^e^ 20.72 ± 1.15	^e^ 21.78 ± 0.99	^d^ 16.46 ± 0.25
50	^d^ 17.52 ± 1.69	^e^ 21.3 ± 2.66	^bcd^ 15.56 ± 2.71
75	^cd^ 15.79 ± 2.69	^e^ 21.54 ± 3.04	^d^ 16.84 ± 1.93
100	^ab^ 12.68 ± 0.77	^bcd^ 15.27 ± 2.01	^bc^ 13.26 ± 0.41

The letters a–f in the upper index indicate belonging to homogeneous groups, between which no statistically significant differences were found (*p* < 0.05).

**Table 8:** Average solubility values in water [%] in respect of the gelatin (0% broth) and gelatin–broth edible films.

Solubility in Water ± Standard Deviation [%]			
Broth Concentration [%]	Gelatin Concentration [4%]	Gelatin Concentration [8%]	Gelatin Concentration [12%]
0	100	^a^ 42.84 ± 0.99	^ab^ 43.14 ± 0.76
25	^bc^ 46.32 ± 2.69	^a^ 42.26 ± 0.30	^a^ 41.94 ± 0.82
50	^cd^ 47.96 ± 0.51	^de^ 50.92 ± 1.19	^c^ 46.88 ± 0.31
75	^de^ 51.15 ± 0.34	^e^ 51.42 ± 0.36	^c^ 47.25 ± 6.22
100	^c^ 46.88 ± 0.60	^e^ 53.47 ± 2.89	^e^ 52.64 ± 0.52

The letters a–e in the upper index indicate belonging to homogeneous groups, between which no statistically significant differences were found (*p* < 0.05).

## Data Availability

Data are contained within the article.
